# A Case of Successful Treatment of Left Ventricular Rupture after Transcatheter Aortic Valve Implantation

**DOI:** 10.15388/Amed.2024.31.2.3

**Published:** 2024-12-04

**Authors:** Rustem Tuleutayev, Kuat Abzaliyev, Alexey Kolesnikov, Igor Kim, Symbat Abzaliyeva

**Affiliations:** 1 Department of Cardiac Surgery, Research Institute of Cardiology and Internal Diseases, Almaty, Republic of Kazakhstan; 2 Consultative and Diagnostic Center, Research Institute of Cardiology and Internal Diseases, Almaty, Republic of Kazakhstan Department of Clinical Specialties, Al-Farabi Kazakh National University, Almaty, Republic of Kazakhstan; 3 Department of X-ray Endovascular Surgery, Scientific Research Institute of Cardiology and Internal Diseases, Almaty, Republic of Kazakhstan; 4 Intensive Care Unit, Research Institute of Cardiology and Internal Diseases, Almaty, Republic of Kazakhstan; 5 Higher School of Medicine, Faculty of Medicine and Healthcare, Al-Farabi Kazakh National University, Almaty, Republic of Kazakhstan

**Keywords:** TAVI, transcatheter aortic valve implantation, aortic valve, prosthesis dislocation, left ventricular rupture, Raktažodžiai: TAVI, transkateterinė aortos vožtuvo implantacija, aortos vožtuvas, protezo dislokacija, kairiojo skilvelio plyšimas

## Abstract

**Background:**

The article talks about a patient who had nonrheumatic disease of the aortic valve and had a left ventricular rupture during a transcatheter aortic valve implantation (TAVI) procedure.

**Methods:**

The discrepancy between the size of the prosthesis and the size of the native aortic valve (mismatch) led to the need to deploy a second prosthesis, which was dislocated into the left ventricular cavity and led to myocardial damage and the development of tamponade.

**Results:**

As a result of timely cardiac surgery and effective measures of the emergency support service, both prostheses were removed from the left ventricular cavity, the aortic valve was replaced with a “Medtronic Hankock No. 25” biological prosthesis, and the left ventricular myocardial rupture was sutured.

**Conclusions:**

In case of the development of such complications during the transcatheter aortic valve implantation (TAVI) procedure with asystole and cardiac tamponade, it was suggested to conduct cardioplegia (instead of chest compressions).

## Abstract (it)

**Įvadas:**

Straipsnyje rašoma apie pacientą, kuris sirgo nereumatine aortos vožtuvo liga ir kuriam transkateterinės aortos vožtuvo implantacijos (TAVI) procedūros metu plyšo kairysis skilvelis.

**Metodai:**

Dėl protezo dydžio ir aortos vožtuvo dydžio neatitikimo reikėjo įdėti antrą protezą, jis buvo dislokuotas į kairiojo skilvelio ertmę ir sukėlė miokardo pažeidimą bei tamponados išsivystymą.

**Rezultatai:**

Dėl laiku atliktos kardiochirurginės operacijos ir veiksmingų skubios pagalbos tarnybos priemonių abu protezai buvo pašalinti iš kairiojo skilvelio ertmės, aortos vožtuvas pakeistas „Medtronic Hankock No. 25“ biologiniu protezu, o kairiojo skilvelio miokardo plyšimas susiūtas.

**Išvados:**

Pasireiškus tokioms komplikacijoms transkateterinės aortos vožtuvo implantacijos (TAVI) procedūros metu su asistolija ir širdies tamponada, pasiūlyta atlikti kardioplegiją (vietoj krūtinės ląstos paspaudimų).

## Background

With increasing life expectancy in economically developed countries, the prevalence of aortic stenosis (AS) complicated by calcinosis in the elderly is a frequent pathology. In a general population, it makes up 2.5% [1]. The prevalence of aortic stenosis is increasing by about 10% among patients over 80 years of age [2]. And in a population over 75 years old, 1 in 8 has moderate or severe aortic stenosis [3]. Whereas pathological changes of the aortic valve (AV) in elderly patients are detected in about 40% of patients, and in octogenarians, similar calcium salt deposits occur in 75% of cases [4]. It is known that in 80% of asymptomatic patients with severe AS, the condition manifests suddenly with symptoms of angina and hypertension. According to the literature, the two-year survival rate for such patients fluctuates around 50%, and when symptoms appear, the survival rate, according to some data, falls to 15% within five years [5]. Almost all patients in this age group have severe comorbidities. These patients have an increased risk of complications and adverse outcomes from AV replacement surgery. Therefore, transcatheter aortic valve implantation (TAVI) in elderly patients with comorbid conditions is the treatment of choice. But in the process of mastering this technique and introducing transcatheter methods into the practice of valve replacement, there were still many questions [5,6]. The negative outcome within a month in elderly patients was as high as 17%, and various complications after 3–4 months brought this figure up to 60% (during the period of mastering the technique) [7,8]. However, complications and mortality rates have significantly decreased with more experience and improvements in medical devices, medicines, and anaesthesia methods. According to recent data, mortality in the immediate postoperative period after TAVI does not exceed 6% [9], and the survival rate within a month after TAVI surgery was 92.9%. Other complications in the same patients result in a 78.6% survival rate after one year and 73.7% after 2 years [10]. Current data show that TAVI significantly reduces financial losses in treating these patients, as well as complications with postoperative mortality [11,12]. Given the study of all complications, causes of mortality, and risks of the procedure, various associations have issued numerous regulations and guidelines for TAVI [13]. Some things that can make complications more likely are a small sinotubular junction and fibrous ring, a very high systolic gradient because the fibrous ring is calcified, and strong predilation.

Typical complications of TAVI are paravalvular leak (PVL) leading to paraprosthetic regurgitation, valve dislocation, complete heart block, left ventricular rupture or aortic dissection, obstruction or thrombosis of coronary arteries with the development of myocardial infarction, embolic complications in the form of strokes, tamponade, and acute renal failure [14]. The complications of TAVI can be classified as intraoperative and postoperative, despite the sparing technique compared to open surgery with the use of artificial circulation. According to T.E. Imaev, A.E. Komlev, M.A. Saidov et al. [15], fibrous ring rupture was observed in 0.4–0.6% of cases, and intraoperative mortality reached 2.6%.

According to life-threatening indications, life-threatening complications require immediate surgery under bypass circulation [16]. Non-life-threatening complications after TAVI are largely determined by the diameter and material of the valve delivery system and include such risks as dissection, rupture, perforation, or total occlusion of iliac or femoral arteries and occur in 15.9% of patients [17]. Within one month after TAVI, the use of a small-diameter valve delivery system reduces such complications to 5.2%–5.9% [18,19]. The incidence of stroke is 2–5% within one month after TAVI [20]. At the same time, dyscirculatory encephalopathies after TAVI, according to brain MRI, were detected in 84% of patients who underwent percutaneous intervention, while the use of artificial circulation revealed 48% of subclinical manifestations of circulatory disorders in the brain [21]. The available literature describes cases of dislocation of prostheses, primarily in the ascending aorta [22, 23]. The authors point out that, due to the complexity of the transcatheter aortic valve replacement procedure, complications are possible even for experienced operators.

This is a clinical case that demonstrates the successful treatment of an intraoperative complication in a patient with nonrheumatic aortic valve disease during TAVI. Due to the discrepancy between the size of the first TAVI prosthesis and the size of the native aortic valve, a significant PVL was registered on Echo. After careful assessment, it was decided to deploy a second TAVI valve of a bigger size. During the deployment of the second valve, the first prosthesis dislocated into the LV cavity, causing damage to the myocardium.

## Case presentation

Patient B., 70 years old, was admitted to the hospital with the following diagnosis: Acquired heart valve disease (nonrheumatic). The patient had a combination of aortic disease and predominant critical stenosis (Sao-0.5 cm^2^). The patient presented with complaints of pressing pain in the retrosternal region with physical exertion and at rest (FC III-IV), shortness of breath, and general weakness. The patient’s medical history indicates that they have had arterial hypertension for over a decade, reaching a maximum blood pressure of 200/80 mm Hg, and have consistently received optimal medical care. Chest pain first appeared 3 years ago; symptoms of heart failure, shortness of breath, and weakness have begun to progress since then. There is no history of myocardial infarction. Suffers from bronchial asthma for a long time and regularly takes bronchodilators.

**Figure 1:**
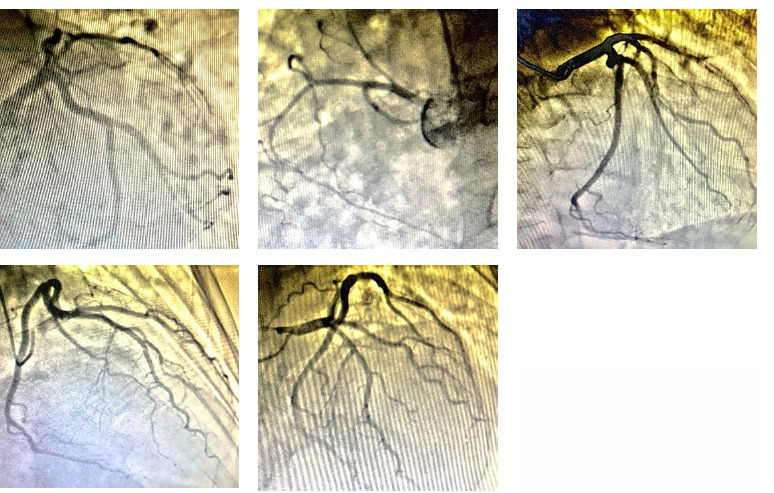
On coronary angiography, a pronounced left type of coronary circulation with a poorly developed right coronary artery (No coronary artery disease was detected).

The patient’s condition has been worsening since October 2020, when the pressure, chest pain, and dyspnoea from physical exertion began to bother him more often. Between October 19, 2020, and October 28, 2020, he was hospitalized with the diagnosis of coronary heart disease (CHD). Braunwald classified his angina as unstable and functional class III. Grade III aortic valve stenosis. The patient has a second-degree tricuspid valve insufficiency. Pulmonary hypertension. CHF with a preserved EF of 66% falls under class III (NYHA). The patient has second-degree arterial hypertension with a risk score of 4. Coronaroangiography on October 24, 2020. Left-type coronary blood flow. The LMCA’s trunk was clear, with a smooth contour. The LAD was clear throughout. AI – all the way through. All the way through, the CX was passable. RCA: clear, small caliber (Figure 1).

The echocardiography test from October 20, 2020 revealed sclerosis and dilatation of the ascending aorta, AV calcification, critical stenosis, and AV insufficiency due to an increase in the gradient on the AV. LV myocardial hypertrophy. LV diastolic dysfunction type II. The LV myocardium’s contractility is reduced. Moderate PH. On November 23, 2020, the patient was hospitalized at the Research Institute of Cardiology and Internal Medicine in the interventional cardiology department for elective transcatheter aortic valve replacement.

**Figure 2:**
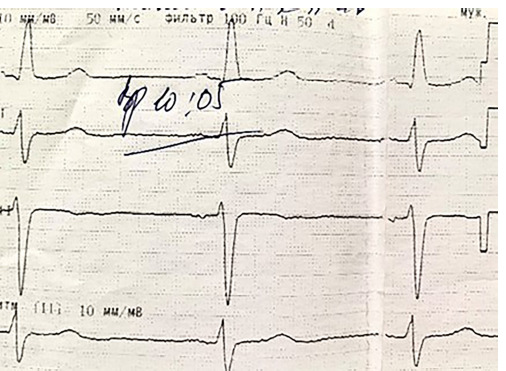
ECG on admission 23.11.2020.

Laboratory and diagnostic tests revealed an increase in blood creatinine of 138 μmol/l. PCR for COVID-19 from November 18, 2020: negative. The department conducted inpatient instrumental and diagnostic tests, including a 6-minute walk test on November 23, 2020, which measured 105 meters. The patient’s ECG showed sinus bradycardia upon admission on November 23, 2020. The patient experienced sinus bradycardia, registering a heart rate of 52 beats per minute. The cardiac electric axis is deviating sharply to the left. There is a blockage in the anterior-superior branch of the left bundle (Figure 2).

## Management and Outcome

**Figure 3:**
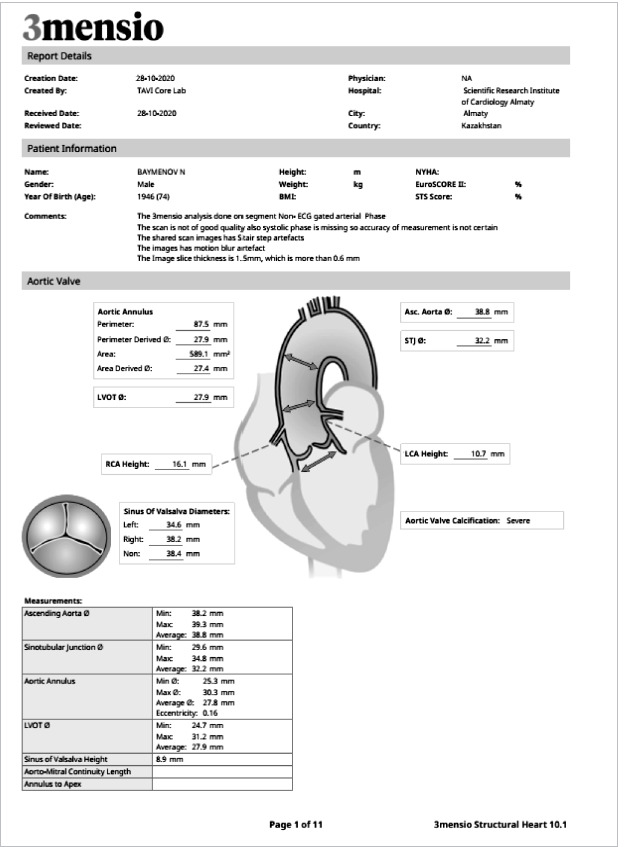
Data results A.

**Figure 4:**
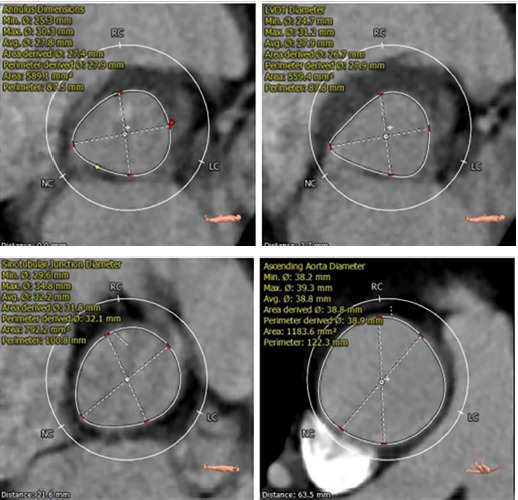
Data results B.

**Figure 5:**
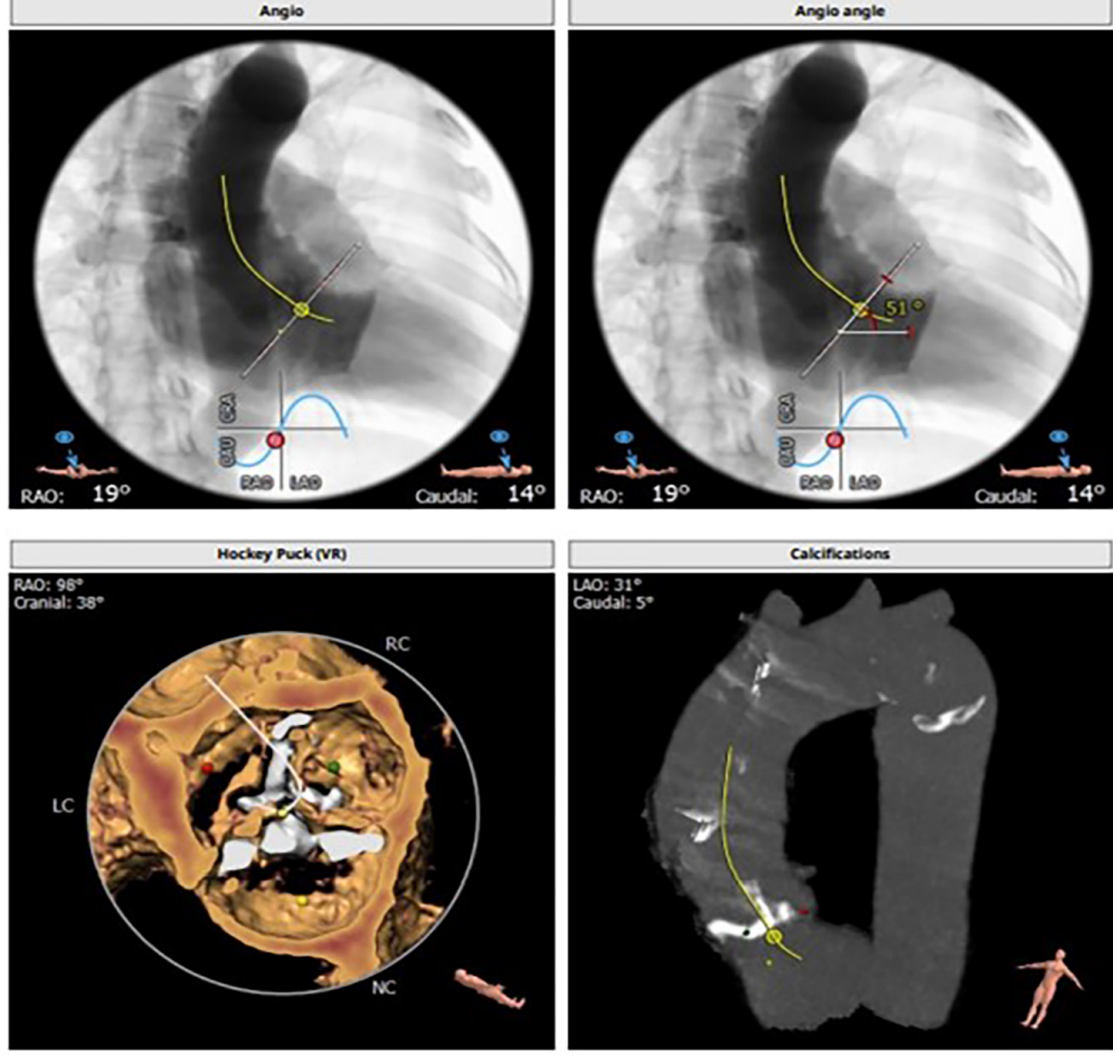
Data results C.

Considering the high risk of open-heart surgery due to the patient’s age, concomitant pathology of the lungs and kidneys, and the presence of symptoms of critical aortic stenosis, the multidisciplinary team decided to perform transcatheter implantation of the MYVAL 27.5 mm aortic valve. The preliminary size of the implantable prosthesis was calculated based on the data of the CT scan with reconstruction and the obtained measurements (Figures 3–5).

**Figure 6:**
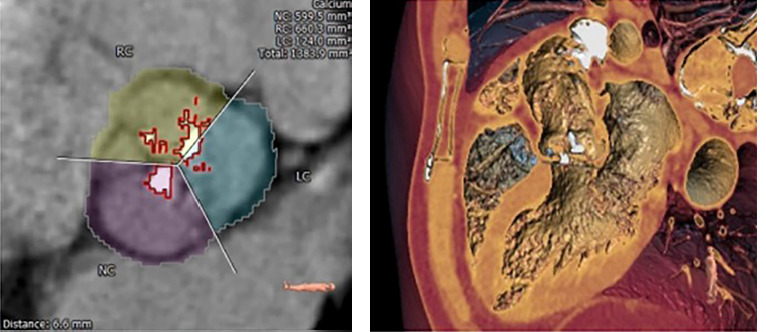
The MYVAL 27.5mm valve was implanted and paraprosthetic contrast delivery into the left ventricle was registered.

The patient underwent surgery. On November 24, 2020, the patient underwent endovascular aortic valve replacement. Procedure progress: We treated the puncture area three times with betadine and once with alcohol. The arterial access was transfemoral on the left; a pigtail catheter was inserted into the noncoronary sinus, venous on the left through the CFV, and for temporary pacing, the electrode was inserted into the RV cavity. Under general anaesthesia, the right common femoral artery was isolated. A 6F intramedullary tube was placed in the arterial lumen. Using an AL II catheter, a straight guidewire was guided into the left ventricle through the stenosed AV orifice. The guidewire was then replaced with an Amplatzer Super Stiff. A 14-Fr intramedullary tube was inserted into the femoral artery lumen. Predilation of the AV was performed (Baloon 20×40mm) for 45 seconds. The aortic valve delivery system was inserted into the aortic root by the guidewire. MYVAL 27.5 mm valve implantation was performed under the fluoroscopy control with simultaneous RV stimulation of 170 beats/min. The control aortography and echo showed a 2- to 3-degree paravalvular leak (Figure 6).

**Figure 7:**
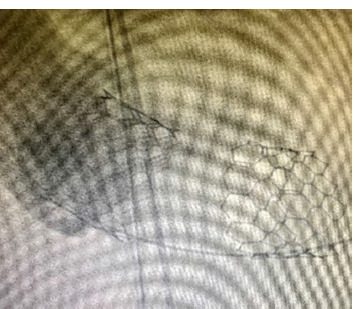
Migration of the first valve into the left ventricular cavity during the installation of the second valve.

Due to the presence of a PVL, it was decided to implant a larger MYVAL 29 mm valve inside the first valve. The technique of placing a larger prosthesis into a small size was used – stretching the nitinol mesh of the stent by ballooning. A guidewire (delivery device) was inserted for the second valve to perform valve-to-valve implantation. Problems with getting the delivery device into the left ventricle happened during the delivery of the second valve. This made it easier for the first valve to move into the left ventricular outflow tract (Figure 7) during the implantation of the second valve.

During the second prosthesis implantation, there was a sharp drop in BP to 60/40 mmHg. Asystole on an ECG monitor. On the screen, there are indicators of contrast agent effusion into the pericardial space. Resuscitation was immediately initiated. Cardiac tamponade was confirmed under TEE. Emergency pericardiocentesis and autohemotransfusion were performed. Cardiac surgeons were informed, and the patient was urgently transferred to the cardiac surgical unit.

Urgently, with continuous chest compression and mechanical ventilation through the Ambu bag, the patient was delivered to the operating unit, intubated, and connected to the ventilator. After treatment of the operating field with povidone iodine, a longitudinal sternotomy was performed without haemostasis. During the revision, hemopericardium and ventricular fibrillation were noted, along with a transmural defect measuring 2x3 cm on the anterolateral surface of the left ventricle, located between the diagonal branches of the anterior descending artery. Within a few seconds, the ascending aorta and right atrium were cannulated with a two-level cannula, “torpedo blood circulation”. Selectively, the left coronary artery underwent cardioplegia, “Kustadiol”. The right superior pulmonary vein provided drainage to the left ventricle. An attempt to suture the defect with parallel perfusion was unsuccessful. A suture eruption was observed. Implanted MYVAL 29 mm valve removed.

**Figure 8:**
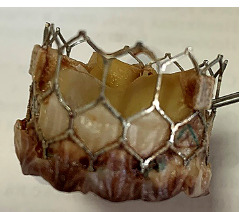
Removed bioprosthesis for TAVI – MYVAL 27.5mm.

On November 24, 2020, from 3:05 to 6:50 p.m., during surgery, the surgeon replaced the aortic valve with a “Medtronic Hankock No. 25” biological prosthesis using bypass circulation. The surgeon sutured the defect in the anterolateral wall of the left ventricle. The transcatheter valve from the left ventricle was removed (Figure 8). The CATS blood-sparing system was installed.

**Figure 9:**
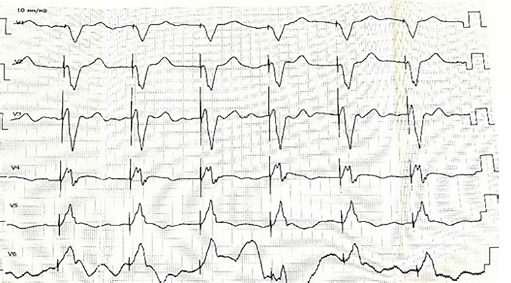
ECG from 24.11.2020: ECS rhythm, clearly imposed, HR 86 bpm.

The native valve leaflets were fibrotic, calcified, and had an incomplete closure. The right coronary cusp ruptured. The aortic valve was dissected. The defect in the anterolateral wall region of the left ventricle was repaired with a double-row suture (mattress + coiled) and Prolene 3.0 suture using xenopericardial strips. A Medtronic Hankock No. 25 biological prosthesis was implanted in the aortic position with 15 U-shaped sutures. Double-row continuous suture to the aorta (Prolene 4/0). Prevention of air embolism. The clamp was removed from the aorta. Myocardial electrodes were sutured. In the early postoperative period, the patient developed a complete atrioventricular block, and a temporary pacemaker was installed. The rhythm is imposed by the pacemaker with a heart rate of 80 per minute (Figure 9).

Once the hemodynamics stabilize, the IC ends. Decannulation. Hemostasis. Installed drains in the anterior mediastinum and the left pleural cavity. The sternum is osteosynthesized. Sutures are used for subcutaneous fat and skin. An aseptic bandage was applied. The drains are connected to the blood-saving CATS system. The patient was transferred to the ICU on an imposed rhythm.

Echo from November 25, 2020: poor ultrasound window. Visualization is extremely difficult. In the dynamics of EF, 43%. The fluid level in the pericardium exceeds 1.0 cm along the inferior wall, with a maximum pressure of 16–18 mm Hg. X-ray from November 29, 2020: At the time of the test, there was mild congestion in the root zones. Cardiomegaly. The aorta has atherosclerosis. The right side exhibits signs of pleural effusion. The CT scan from December 7, 2020 revealed signs of a left-sided pleural effusion. The lower lobe of the left lung exhibits atelectasis. Left-sided lower lobe pneumonia. Congestive changes in the lungs. Operated heart. A complete transverse blockade persisted for 18 days after the aortic valve implantation, after which the independent sinus rhythm was restored. The temporary pacemaker was removed. In the follow-up X-ray of the OGK, the signs of pneumonia, atelectasis, and exudative pleurisy had resolved. In the control examination for echocardiography on AC, there is no pathological gradient, regurgitation of 1 degree, and an EF 50%.

In the postoperative period, the patient developed neurological disorders due to a transient ischemic attack. No complications were observed from the side of the postoperative wound or the implanted prosthesis. The patient was transferred to a specialized clinic for follow-up care and the correction of neurological complications. After one month of follow-up, the patient recovered both cognitive and neurological functions. The function of the implanted aortic valve is not impaired. Hemodynamics is unaffected. With the acquisition of experience and the improvement of technical characteristics, the results of TAVI have improved. However, it is necessary to consider not only the patient’s clinical status but also the issues of organizing and managing the process.

## Discussion

The likelihood of developing such a complication is low, but it exists [22,23]. If the size of the valve is correctly calculated, taking into account the fact that a PVL may occur, it is possible to repeat the inflation of the balloon with high pressure. If the valve is mismatched with a small diameter, then paravalvular leaks will always develop, and the probability of valve dislocation is very high, which happened in this case.

Thus, from the literature sources and having studied the remaining documents during the analysis of the medical records, the following conclusions were made.

1. The dislocation of the aortic valve was apparently due to a lack of good fixation in the fibrous ring of the aortic valve and a mismatch between the size of the prosthesis and the fibrous ring [24].
2. Cardiac tamponade and LV rupture occurred when the second valve was inserted during the delivery system, which pushed the first prosthesis into the left ventricular cavity and damaged the anterior wall of the left ventricle [25].
3. Damage to the left ventricle with the first prosthesis and ongoing resuscitation measures, including chest compressions, led to further traumatic rupture of the left ventricle’s myocardial wall [26].
4. Effective resuscitation measures and timely delivery of the patient to the operating room allowed the team of cardiac surgeons to successfully replace the aortic valve, remove the TAVI valve, and suture the rupture of the LV wall [27].
5. Transient neurological complications arising after surgery are associated with large blood loss and impaired cerebral circulation during resuscitation measures [28].
6. A well-organized, interdisciplinary service contributed to the positive outcome in this case [29].

Based on the above, the following recommendations. It is necessary to very carefully calculate the size of the prosthesis to be implanted in order to avoid the development of paravalvular leaks (fistulas). When installing the second prosthesis, carrying out the delivery system requires caution and careful control to avoid dislocation of the first valve into the left ventricular cavity. When carrying out resuscitation measures, especially chest compressions, in the presence of a delivery system (with a prosthesis) in the left ventricular cavity, it is necessary to take into account the risk of developing a traumatic myocardial rupture. Consider the possibility of urgent removal of the delivery system and selective cardioplegia with the introduction of the patient into anesthesia, cerebral hypothermia, and mechanical ventilation. It is recommended to perform transcatheter aortic valve implantation in the presence of cardiac surgical support.

## Conclusion

TAVI is an increasingly utilized procedure for treating severe aortic stenosis in high-risk patients. While generally safer than open surgical aortic valve replacement, TAVI still carries significant risks of complications, some of which can be life-threatening emergencies.

This case highlights the potential for catastrophic complications like valve dislodgement, ventricular rupture, and cardiac tamponade to occur even in experienced centres. Precise sizing to avoid prosthesis–patient mismatch and careful valve deployment technique is critical to prevent such disasters. When they do occur, rapid interdisciplinary response involving emergency resuscitation, pericardiocentesis, and prompt transfer to cardiac surgery is essential for patient survival. Avoidance of chest compressions during resuscitation for cardiac arrest in the setting of an indwelling transcatheter valve may also be prudent to prevent further myocardial injury. Selective administration of cardioplegia and cerebral protection rather than conventional ACLS may be a better temporizing approach preoperatively.

While serious complications like this patient experienced are uncommon, their rapid recognition and treatment require a true multidisciplinary systems-based approach to optimize outcomes. As TAVI continues expanding to lower-risk patients, meticulous procedural planning and backup emergency pathways become even more paramount.

## Data Availability

The authors confirm that the data supporting the findings of this study are available in the article.
